# A CaCuSi_4_O_10_/GCE electrochemical sensor for detection of norfloxacin in pharmaceutical formulations†

**DOI:** 10.1039/d3ra01702h

**Published:** 2023-04-25

**Authors:** Gregarious Muungani, Werner E. van Zyl

**Affiliations:** a School of Chemistry and Physics, University of KwaZulu-Natal Westville Campus Durban 4000 South Africa vanzylw@ukzn.ac.za +27 31 260 3199

## Abstract

This study reports on a calcium copper tetrasilicate (CaCuSi_4_O_10_)/glassy carbon electrode (GCE) electrochemical sensor developed for rapid sensing and quantification of an antibacterial drug, norfloxacin, using both cyclic voltammetry and differential pulse voltammetry. The sensor was fabricated by modifying a glassy carbon electrode with the CaCuSi_4_O_10_. Electrochemical impedance spectroscopy was performed and the Nyquist plot showed that the CaCuSi_4_O_10_/GCE had a lower charge transfer resistance of 22.1 Ω cm^2^ compared to the GCE with a charge transfer resistance of 43.5 Ω cm^2^. Differential pulse voltammetry showed that the optimum pH for the electrochemical detection of norfloxacin in potassium phosphate buffer solution (PBS) electrolyte was pH 4.5 and an irreversible oxidative peak was found at 1.067 V. Two linear ranges were established at 0.01 to 0.55 μM and 0.55 μM to 82.1 μM, and the limit of detection was *ca.* 0.0046 μM. We further demonstrated that the electrochemical oxidation was controlled by both diffusion and adsorption processes. The sensor was investigated in the presence of interferents and was found to be selective toward norfloxacin. The pharmaceutical drug analysis was done to establish method reliability and a significantly low standard deviation of 2.3% was achieved. The results suggest that the sensor can be applied in the detection of norfloxacin.

## Abstract 2

A calcium copper silicate (CaCuSi_4_O_10_)/glassy carbon electrode (GCE) sensor was developed for sensing of an antibacterial drug, norfloxacin, using both cyclic voltammetry and differential pulse voltammetry.

## Graphical abstract

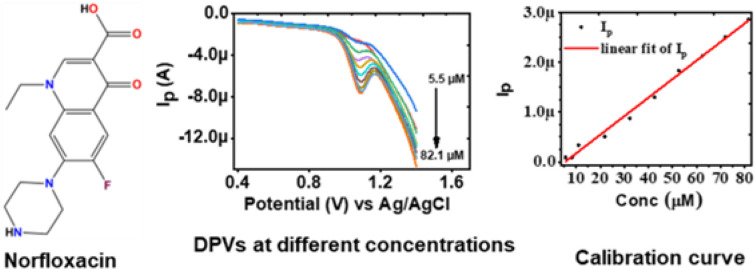

## 1 Introduction

Norfloxacin (NFX), 1-ethyl-6-fluoro-1,4-dihydro-4-oxo-7-(1-piperazinyl)-3-quinolone carboxylic acid, is a synthetic fluoroquinolone^1^ that is active against Gram-positive and Gram-negative bacteria and is widely used to treat both urinary and respiratory tract infections.^2,3^ The drug is used to treat both human and veterinary infections and it acts by inhibiting the synthesis of bacterial DNA gyrase and bacterial chromosome replication.^4^ The widespread usage of norfloxacin coupled with the need for clinical and pharmacological studies necessitated the development of sensitive and rapid analytical methods for its quantitative determination.

Following the use of norfloxacin, its presence was found in municipal wastewater treatment plants, hospital wastewater, surface, and ground waters at nano and micro levels.^5^ However, the accumulation of norfloxacin in water results in antibiotic-resistant bacteria, it also has the potential to disrupt the endocrine of aquatic organisms, and reduces the formation of colonies in *Scenedesmus quadricauda*; thus, becomes vulnerable to zooplankton grazers.^6^ Furthermore, norfloxacin presence in the environment inhibits the growth of denitrifying bacteria and the activity of its enzymes.^7^ Hence, there is a need to monitor the presence of norfloxacin in the environment so that appropriate countermeasures aimed at minimising its pollution into the environment can be implemented.

Methods such as liquid chromatography-tandem mass spectrometry (LC-MS),^8^ fluorescence-spectrophotometry,^9^ electro-chemiluminescence,^10^ capillary electrophoresis,^11^ high-performance liquid chromatography (HPLC)^12^ and spectrofluorimetry^13^ were used to analyse for the presence of norfloxacin. However, some of these methods are time-consuming and require laborious sample pre-treatment, large organic solvent consumption, expensive equipment, and advanced technical expertise. In this study, electrochemical techniques were used for the detection of norfloxacin in pharmaceutical formulation. The advantages of electrochemical methods include being simple to operate, relatively cost-effective and sensitive, having a rapid response speed; thus, offering real-time detection.^14^ This method aims to provide both good sensitivity and a wide linear range in the determination of norfloxacin.

Herein we report on the use of a CaCuSi_4_O_10_ modified glassy carbon electrode (CaCuSi_4_O_10_/GCE) for electrochemical sensing of norfloxacin. CaCuSi_4_O_10_ is a layered clay mineral, a phyllosilicate that naturally exists,^15^ it is abundant, and can also be synthesized. A phyllosilicate is a layered silicate^16^ that has a structure made up of ions (or atoms), which are arranged in sets of parallel planes bonded together to form layers.^17^ The layers may be charge-balanced, *i.e.* neutral, or may have charge^17–20^ making redox reactions necessary for electrochemical detection possible. Additionally, the layered silicates have interlayer space available for adsorption^21^ and these combined attributes as it relates to CaCuSi_4_O_10_ in particular can be leveraged for the electrochemical detection of drugs such as norfloxacin. The transition metal oxide MnO_2_ was added to CaCuSi_4_O_10_ to improve electrical conductivity.

## 2 Experimental section

### 2.1 Chemicals and materials

Analytical reagents used in this study had a purity ≥98%. Calcium chloride, copper nitrate, tetraethyl orthosilicate (TEOS), ammonium hydroxide solution (25 wt%), ethanol, potassium ferricyanide, potassium ferrocyanide, potassium dihydrogen phosphate, dipotassium hydrogen orthophosphate, norfloxacin were obtained from Merck, Germany. Utin-400 (Cipla, Durban, South Africa) was purchased from a local pharmacy. Monopotassium dihydrogen orthophosphate and dipotassium hydrogen orthophosphate were used in the preparation of 0.1 M phosphate buffer solutions of different pHs for the pH study. Double distilled water was used throughout the experiment.

### 2.2 Instruments

Powder X-ray diffraction (PXRD) analysis was done using an X-ray diffractometer (Bruker AXS D8 Advance, Germany), equipped with a Cu-Kα radiation source (wavelength = 0.154 nm) operating at 40 kV and 40 mA. The XRD pattern was recorded over the angular range 2*θ* = 5–90° at room temperature. Surface topography and composition of the samples were performed by a Zeiss Ultra Plus Field Emission Gun Scanning Electron Microscope (FEG-SEM) equipped with an energy-dispersive X-ray (EDX) detector (Germany). A JEOL 1010 (Japan) Transmission Electron Microscope (TEM) with a JEOL 2100 (Japan) High-Resolution Transmission Electron Microscope (HRTEM) was used to obtain TEM micrographs. Raman spectra of the samples were done using a DeltaNu Advantage 532 high-performance Raman spectrometer with a resolution ranging from 8–10 cm^−1^, and a spectral range of 200–3400 cm^−1^. The spectrometer used a 532 nm solid-state frequency-doubled Nd:YAG laser with a peak power of 200 mW and a 35 μm diameter focused beam. Infra-red spectroscopic analysis was done by a Spectrum 100 infrared spectrometer equipped with a universal diamond crystal attenuated total reflection (ATR) accessory (PerkinElmer, USA) within the wavenumber range 380–4000 cm^−1^ at a resolution of 4 cm^−1^. A Hanna Instruments pH meter (Woonsocket RI, USA) with a glass electrode was used to obtain pH measurements.

The CHI 660E electrochemical workstation (USA) was used for electrochemical studies. Electrochemical measurements were done using a three-electrode setup comprising a Ag/AgCl electrode (reference electrode), glassy carbon electrode (GCE) (working electrode, diameter 3 mm), and platinum wire (counter electrode). Electrochemical impendence spectroscopy (EIS) measurements were performed at a frequency range of 10 000 Hz to 1 Hz with 5 mV amplitude. A supporting electrolyte of both 2.5 mM [Fe(CN)_6_]^3−^ and 2.5 mM [Fe(CN)_6_]^4−^ in 0.1 M KCl was used. For differential pulse voltammetry (DPV), 50 mV pulse amplitude and 50 ms pulse width were used while varying the step potential (3–8 mV).

### 2.3 Synthesis of CaCuSi_4_O_10_

CaCuSi_4_O_10_ was synthesized in aqueous medium through a one-pot Stöber sol–gel synthesis of silica^22^ followed by co-precipitation of calcium and copper metal precursors. TEOS and the calcium and copper metal precursors were added according to their mole ratios in CaCuSi_4_O_10_, which is 1 : 1 : 4 for calcium, copper and silica, respectively. The precipitate was filtered, rinsed three times with double distilled water, dried in an oven for 12 h at 120 °C and heated in muffle furnace at 800 °C for 24 hours.^23,24^

### 2.4 Fabrication of the CaCuSi_4_O_10_ modified glassy carbon electrode

Initially, the GCE was cleaned with alumina slurry to polish its surface and then rinsed with deionised water to remove any adsorbed alumina particles. Afterward, a 1 mg mL^−1^ suspension of CaCuSi_4_O_10_ with 10 wt% MnO_2_ (for conductivity) was prepared and 5 μL was drop cast on the surface of the polished and clean GCE. The electrode was exposed to IR radiation from a lamp for ten minutes to obtain initial dryness, which resulted in a CaCuSi_4_O_10_/GCE modified electrode. The modified electrode was used for norfloxacin drug sensing electrochemical measurements using a CHI660E electrochemical workstation. Measurements were done in replicates of three and for each run, the GCE was freshly coated with the CaCuSi_4_O_10_.

### 2.5 Preparation of pharmaceutical samples

Utin-400 tablet (Cipla) that contained 400 mg norfloxacin was finely ground to powder using a mortar and pestle. The obtained norfloxacin powder was dissolved in distilled water to prepare a suitable stock solution. Differential pulse voltammetry results of the detection of norfloxacin in the presence of pharmaceutical excipients at the CaCuSi_4_O_10_/GCE electrochemical sensor were recorded using diluted aliquots within the linear concentration range.

### 2.6 Electrochemical equations

The following equations were used to calculate specific parameters. The Scherrer equation, eqn (1) (ref. 25)

1 *D*_*hkl*_ = *Kλ*/(*B*_*hkl*_ cos *θ*)

where *D*_*hkl*_ is the crystallite size in the direction perpendicular to lattice planes, *hkl* are the Miller indices of planes of concern, *K* is a dimensionless number of about 0.9, *λ* is the wavelength of X-rays, *B*_*hkl*_ is the full width at half maximum of the X-ray peak in radians and *θ* is the Bragg angle.

Randles–Ševčík equation, eqn (2),^26^

2 *i*_p_ = 2.69 × 10^5^*n*^3/2^*AD*^1/2^*Cν*^1/2^

where *ν* (V s^−1^) is the scan rate, *n* is the number of electrons transferred in the electrochemical process, *i*_p_ (amperes) is the peak current, *C* (mol cm^−3^) is the concentration of the electroactive species, and *D* (cm^2^ s^−1^) is the diffusion coefficient (*D* = 7.69 × 10^−6^ cm^2^ s^−1^) for [Fe(CN)_6_],^3^eqn (3).^27^3
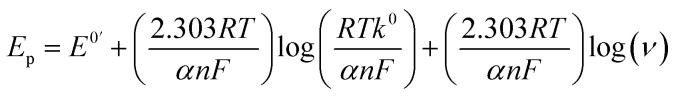
*E*^0^ is the formal electrode potential, *α* is the electron transfer coefficient, *k*^0^ is the standard heterogeneous rate constant, *R* is the universal gas constant = 8.3145 J mol^−1^ K^−1^, *F* is the Faraday = 96 485 coulomb, and *D* is the diffusion coefficient^28^4
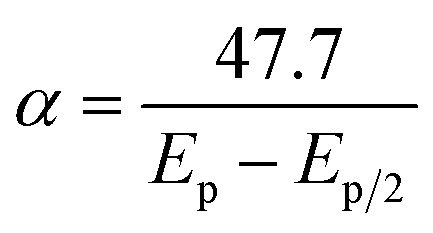
where *E*_p/2_ is the half-peak potential.

## 3 Results and discussion

### 3.1 Physical characterization

**Fig. 1:**
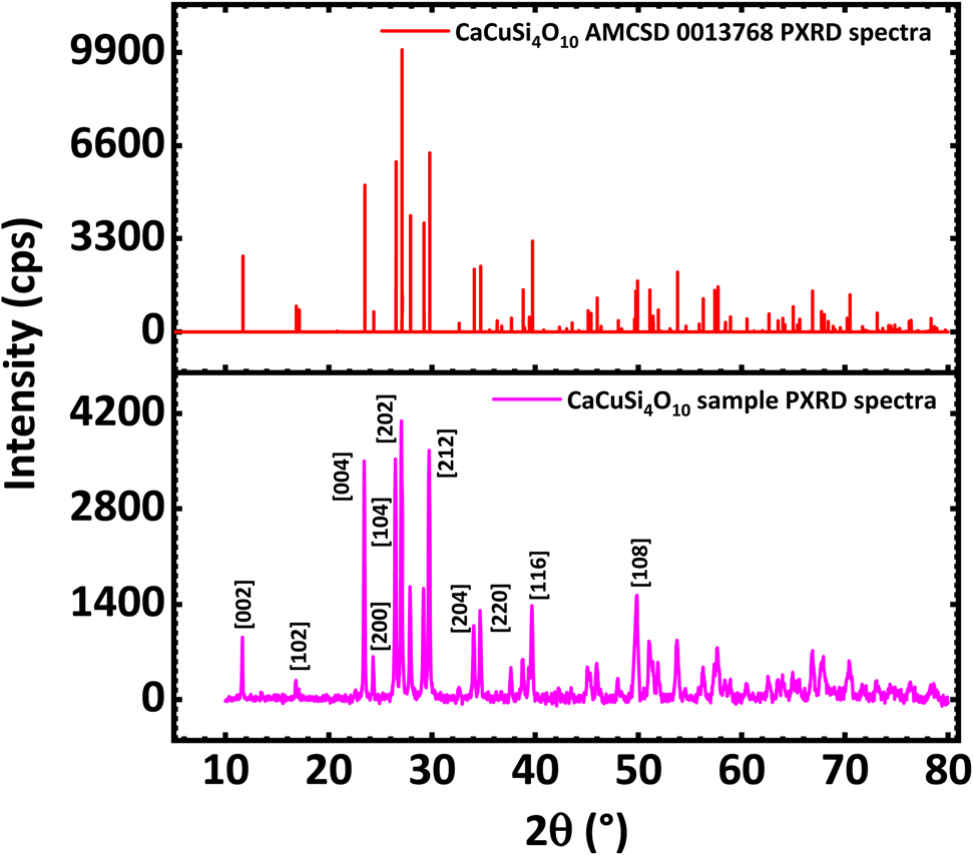
PXRD spectra of CaCuSi_4_O_10_ (below) compared against PXRD of CaCuSi_4_O_10_ (AMC SD 0013768) standard (above).

The PXRD diffractograms of the synthesized CaCuSi_4_O_10_ sample are shown in Fig. 1 and confirmed the successful synthesis as compared to the American Mineralogist Crystal Structure Database reference standard, AMCSD 0013768. The sharp peaks of the PXRD diffractograms indicate the material is crystalline. As shown on the diffractogram of Egyptian blue and also in agreement with the literature,^29^ the pattern can be indexed exactly to the (200) and (020) reflections, which provides a zone axis of [001]. The PXRD data for Egyptian blue also shows basal cleavage along the (001) plane and preferred orientation along the (001) series, which suggested a stacked alignment of such anisotropic particles when drop-cast onto a substrate.^30^ The X-ray diffraction (XRD) pattern of the Egyptian blue shows a ditetragonal–dipyramidal structure.^29^ The CaCuSi_4_O_10_ phyllosilicate belongs to the gillespite series and has a structure belonging to the *P*4/*ncc* – *D*^8^_4*h*_ space group. The maximum diffraction peak showed at 2theta degrees corresponding to 27.04 with a full width at half maximum of 2.88 × 10^−3^ radians. Furthermore, the crystallite size was calculated using the Scherrer equation to obtain 49.46 nm, which reveals that nano-crystallite CaCuSi_4_O_10_ was synthesised. Indications were that the synthesised CaCuSi_4_O_10_ was a probable electroactive material for the electrochemical detection of norfloxacin deriving from its layered structure, properties and the nano-crystallite sized nature, which size is known to facilitate some reactions that may not be possible at a relatively larger physical size.

The FT-IR spectra are shown in Fig. S1.† Notably, the CaCuSi_4_O_10_ sample has intense peaks within the range 980–1055 cm^−1^ due to the silicon–oxygen stretching vibrations. CaCuSi_4_O_10_ has notable bands at 1050, 1000, and 1161 cm^−1^ that is attributed to antisymmetric Si–O–Si stretching vibrations. The vibration modes in the range 1000–1200 cm^−1^ resulted from bond stretching of oxygen relative to silicon, which involves both silicon and oxygen displacements.^31^ Compounds that have tetrahedrally coordinated silicon have a complex absorption band due to Si–O stretching in the range 800–1100 cm^−1^.^32^ The degree of polymerization of the SiO_4_^4−^ tetrahedral determines the actual position of this band.^33^ The 754 cm^−1^ weak IR bands may be attributed to an OH-vibration mode of crystal water.^34^ Peaks below 600 cm^−1^ are mostly attributed to bending vibrations^35^ and are associated with cation–oxygen bending vibrations.^36^ Around 450 cm^−1^ rocking vibrations occur,^37^ which is one of the types of bending vibration together with the scissoring, wagging, or twisting vibrations.^38^ The peaks obtained for the sample at 479 cm^−1^ and 411 cm^−1^ are in agreement with literature reports.^35^ Modes A_2u_ and E_u_ are IR-active and give rise to observed peaks on the IR spectra.

The Raman spectra of CaCuSi_4_O_10_ were obtained as shown in Fig. S2.† Intense peaks at 425 cm^−1^ and 1080 cm^−1^, were observed. According to lattice dynamics, modes A_1g_, B_1g_, B_2g_ and E_g_ are Raman active and give rise to peaks observed for gillespite and its isostructural minerals such as Egyptian blue.^39^

**Fig. 2:**
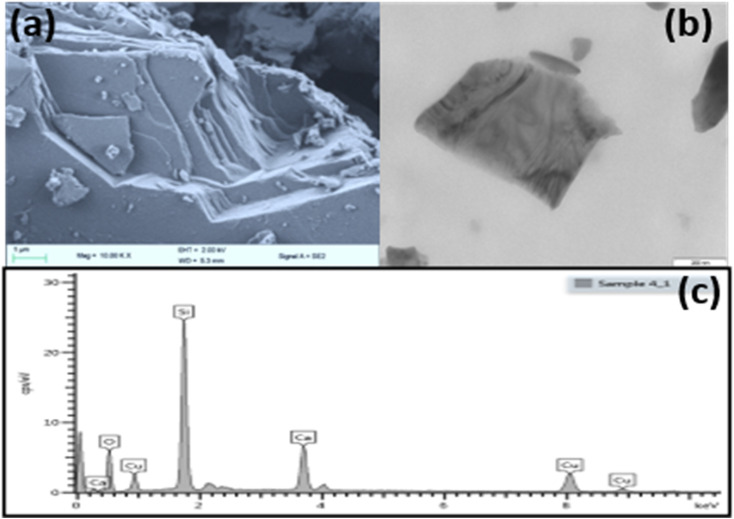
(a) SEM micrograph, (b) TEM and (c) SEM EDX of CaCuSi_4_O_10_.

The SEM and TEM analysis was performed on CaCuSi_4_O_10_ and pictures are presented in Fig. 2(a) and (b). The SEM images confirmed the layered nature of the phyllosilicates while TEM showed a layer of the CaCuSi_4_O_10_. The SEM EDX analysis, Fig. 2(c), illustrated the composition of each of the phyllosilicates. The elements constituting CaCuSi_4_O_10_ were determined by matching the energy values of the recorded distinctive peaks of the obtained X-rays with the associated elements and the particular transition involved. Consistent with previous reports, the peaks at 3.690, 8.040, 1.739 and 0.525 keV corresponded to the Kα line of Ca, Cu, Si and O,^40^ while the peak at 0.930 keV corresponded to the Lα value of Cu.

### 3.2 Electrochemical characterization of electrodes

**Fig. 3:**
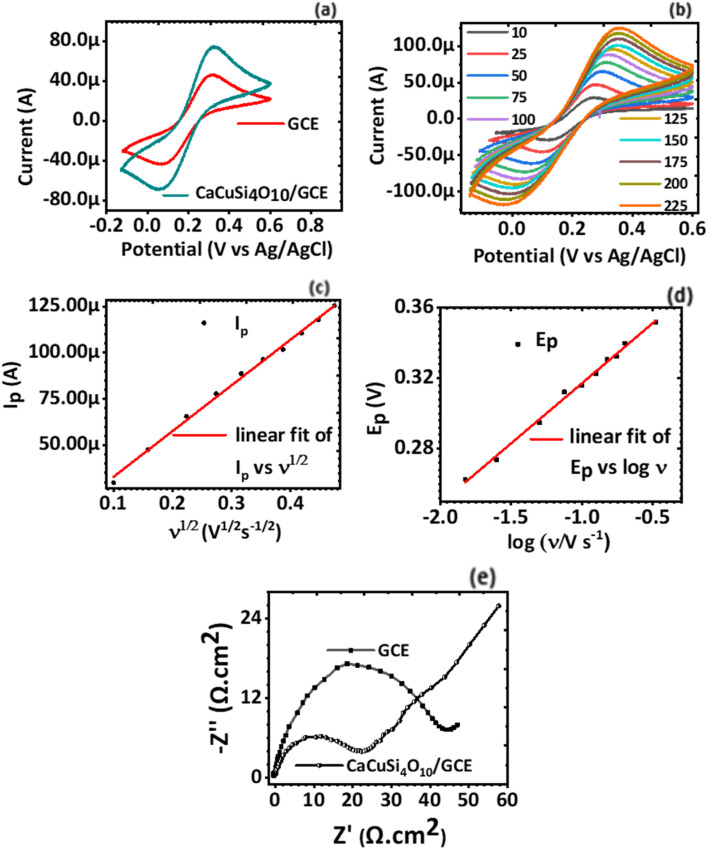
(a) Cyclic voltammograms of GCE and CaCuSi_4_O_10_/GCE, (b) cyclic voltammograms of CaCuSi_4_O_10_/GCE with change in scan rate, (c) plot of *i*_p_*vs. ν*^1/2^ for the CaCuSi_4_O_10_/GCE for various scan rates (0.01 to 0.2 V s^−1^), (d) linear plot of the peak potential *vs.* log scan rate (V s^−1^) and (e) the Nyquist plot of modified and bare GCEs. The electrolyte used was 0.1 M KCl containing KCl containing 2.5 mM [Fe(CN)_6_]^3−^ and 2.5 mM [Fe(CN)_6_]^4−^.

Cyclic voltammetry (CV) was used to establish the electrochemical behaviour, in particular the electron transport properties of the GCE and CaCuSi_4_O_10_/GCE using 2.5 mM [Fe(CN)_6_]^3−^ and 2.5 mM [Fe(CN)_6_]^4−^ as the redox couple probe. The current response to the voltage potential at a scan rate of 100 mV s^−1^ was plotted, Fig. 3(a). The cyclic voltammograms for the GCE and CaCuSi_4_O_10_/GCE electrodes showed well-defined reduction and oxidation peaks due to [Fe(CN)_6_]^3−^ and [Fe(CN)_6_]^4−^. It was noted that the CaCuSi_4_O_10_/GCE modified electrode had a better current response compared to the bare GCE. The better current response for the CaCuSi_4_O_10_/GCE modified electrode was established from the scan rate studies, Fig. 3(b), to be a result of higher electrochemical surface area compared to the bare GCE.

Scan rate studies were done to find out the effect of the scan rate on the electrode current response of the electrochemical system. Furthermore, the active electrochemical surface areas for both the GCE electrode and the CaCuSi_4_O_10_/GCE modified electrodes were deduced using the Randles–Ševčík equation, eqn (2). CVs of the modified GCE electrode in KCl containing 2.5 mM [Fe(CN)_6_]^3−^ and 2.5 mM [Fe(CN)_6_]^4−^ solution were obtained at various scan rates ranging from 10 mV s^−1^ to 200 mV s^−1^. From the raw data, plots of both *i*_p_*vs. ν*^1/2^ (Fig. 3(c)) and *E*_p_*vs.* log *ν* (*E*_p_ is a voltage corresponding to the peak voltage) (Fig. 3(d)) were also performed. It was noted that the anodic current response of the electrochemical system varies linearly with the square root of the potential scan rate. In particular, as the potential scan rate increased, the current response also increased linearly. The relationship was defined by the equation *I*_p_ = 8.2933*μA* + 246.8568*μν*^1/2^, *r* = +0.9981, which shows a linear relationship.

The electrochemical surface area of the CaCuSi_4_O_10_/GCE modified electrode was calculated from the slope of the *i*_p_*vs. ν*^1/2^ (Fig. 3(c)) plot and was found to be 0.0463 cm.^2^ Compared to the electrochemical surface area of the bare GCE (0.0384 cm^2^), the CaCuSi_4_O_10_/GCE modified electrode had a higher surface area. The CaCuSi_4_O_10_/GCE modified electrode, therefore, provided a great interfacial area for the electrochemical oxidation of norfloxacin necessary for electrochemical drug sensing.

A plot of *E*_p_*vs.* log *ν* was done from the raw data of the scan rate studies. It was established that the peak potential (*E*_p_) obtained for each scan rate observed a linear relationship with log *ν* as shown in Fig. 3(d). The linear relationship is defined by the equation *E*_p_ = 0.38544 + 0.06823 log *ν*, where the intercept is 0.38544, the slope is 0.06823, and *r* = +0.99509. There is a linear relationship between *E*_p_ and log *ν* and additionally, a Nyquist plot (Fig. 3(e)) was done from the electrochemical impedance spectroscopy (EIS) data. The charge transfer resistance of the CaCuSi_4_O_10_/GCE is 22.1 Ω cm^2^ and that of the GCE is 43.5 Ω cm^2^. The charge transfer resistance obtained for bare GCE is higher to that obtained for the CaCuSi_4_O_10_/GCE electrochemical sensor, suggesting that comparatively diffusion of [Fe(CN)_6_]^3−^/[Fe(CN)_6_]^4−^ to the CaCuSi_4_O_10_/GCE is more favourable. Thus, to reduce charge transfer resistance of the GCE, it is was necessary to modify the GCE with CaCuSi_4_O_10_. Based on the favorable electrochemical attributes of the CaCuSi_4_O_10_/GCE, subsequent reactions were carried out for this modified electrochemical sensor.

### 3.3 Electrochemical sensing of norfloxacin

#### 3.3.1 pH studies

**Fig. 4:**
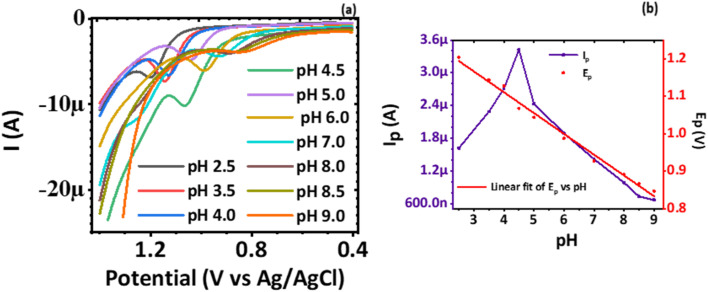
Plot of (a) *I vs. V*, (b) *i*_p_*vs.* pH and *E*_p_*vs.* pH from differential pulse voltammograms of 0.1 mM norfloxacin solution at the CaCuSi_4_O_10_/GCE.

The influence of pH on the electrochemical sensing of norfloxacin was investigated using the differential pulse voltammetry (DPV) technique in the presence of 0.1 mM norfloxacin in potassium phosphate buffer solution (PBS) in a pH range 3–9. The current response to potential is shown in Fig. 4(a). A plot of the current response to pH and a plot of peak voltage (*E*_p_) against pH was done as shown in Fig. 4(b). An optimum pH, where the highest current response was obtained, was established as pH 4.5, where the current was 3.406 μA. The PBS electrolyte at pH 4.5 was used for further studies. The solubility of norfloxacin in water is pH dependent and increases significantly below pH 5, coupled with the fact that its p*K*a1 value is 6.34,^41^ suggesting that the optimum pH for its detection is mildly acidic as obtained in this study, the p*K*a1 value derived from the carboxylic acid group. Norfloxacin also has another p*K*a2 value of 8.47, which is derived from the nitrogen and piperzinyl ring. It was also noted, as shown in Fig. 4(b), that the peak current corresponded to a peak voltage potential of 1.067 V, which concurred with the literature results.^42^ The peak potential, *E*_p_, varied linearly with the pH according to eqn (5),

5 *E*_p_ = 1.33 − 0.05543pH

with 1.33 being the intercept, 0.05543 is the slope and the coefficient of correlation being −0.99484. *E*_p_ had a negative linear relationship with pH. As the pH increased, the peak potential moved to lower values, suggesting the presence of a proton-coupled electron transfer reaction. The linear regression equation eqn (5) can be compared with the Nernst equation, which relates *E*_p_ to pH as in eqn 66
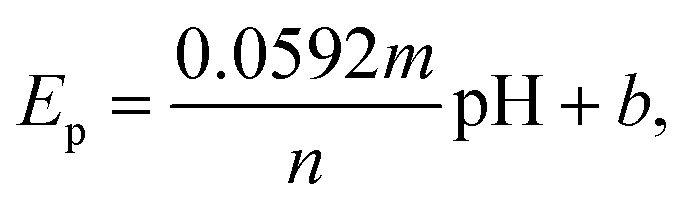
where *n* and *m* represent the number of electrons and protons involved during the electrochemical reaction and *b* is the intercept. A comparison of the slope (0.05543) obtained from the linear regression equation of *E*_p_*vs.* pH of Fig. 4(b) and 0.0592 V/pH from the Nernst equation, eqn (6), shows that the two values are close. The value of *m* equals *n* and the number of protons and electrons that are transferred during the redox reaction of norfloxacin were therefore equal.

#### 3.3.2 Scan rate studies

**Fig. 5:**
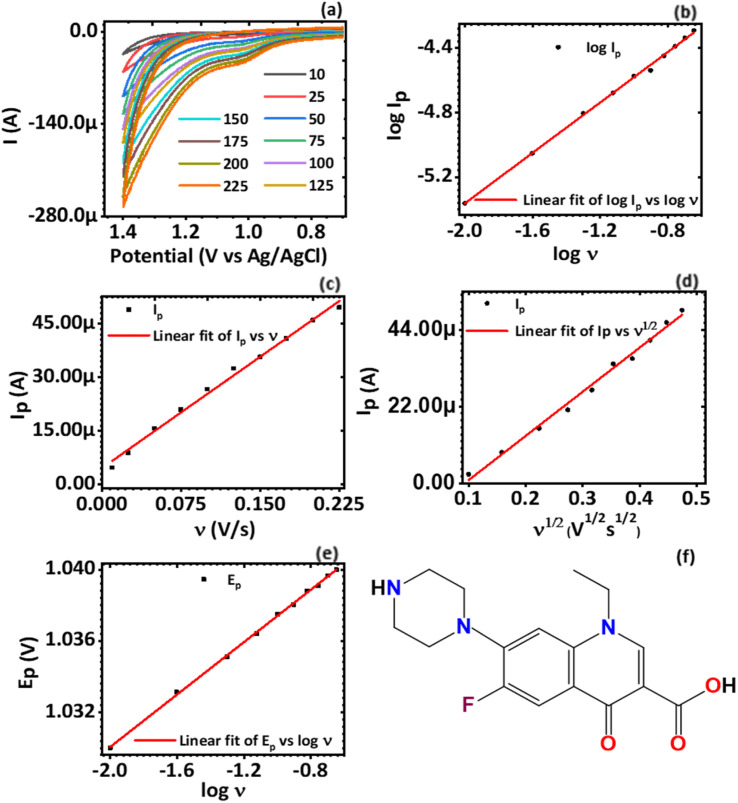
Plot of (a) cyclic voltammograms at scan rates ranging from 10 mV s^−1^ to 250 mV s^−1^, (b) log *I*_p_*vs.* log *ν*, (c) *I*_p_*vs. ν*, (d) *I*_p_*vs.*
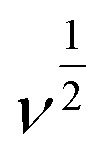
 and (e) *E*_p_*vs.* log *ν* of 0.1 mM norfloxacin at CaCuSi_4_O_10_/GCE modified electrode in PBS pH 4.5 and (f) drawing of norfloxacin.

Cyclic voltammetry technique was used to establish the electrochemical behavior of CaCuSi_4_O_10_/GCE modified GCE electrode towards norfloxacin in the presence of 0.1 M PBS at pH 4.5 by analyzing various scan rates (10–250 mV s^−1^). The investigation was intended to find out the nature of the oxidation process of the norfloxacin at CaCuSi_4_O_10_/GCE modified electrode; that is, if it was diffusion or adsorption controlled. A plot of the current response to the voltage scan (*I*_p_*vs. V*) was done, Fig. 5(a). An oxidation peak was observed at 1.067 V and the peak position is consistent with literature reports.^43^ Furthermore, the cyclic voltammogram showed an irreversible electrode process and for an oxidation peak observed, a corresponding reduction peak was therefore not found.

A plot of log *I*_p_*vs.* log *ν* was also done, Fig. 5(b). It was established that log *I*_p_ varied linearly with log *ν* as defined by the linear regression equation obtained from Fig. 5(b), eqn (7)

7 log *I*_p_ = −3.7526 + 0.7846 log *ν*

where −3.7526 is the intercept, 0.7846 is the slope, and the linear coefficient of correlation is 0.9992. Thus, log *I*_p_ showed a positive linear correlation with log *ν*. The value of the slope (0.7846) is between the theoretical values of 0.5, typical for a redox process controlled only by diffusion mass transport, and 1.0, a value that is for redox processes governed by adsorption.^44^ To establish whether the diffusion or the adsorption process dominated the electron transfer, a plot of peak current (*I*_p_) *versus* potential scan rate (*ν*), Fig. 5(c), and also peak current (*I*_p_) *versus* square-root of potential scan rate (*ν*^1/2^), Fig. 5(d), were plotted. It was found that *I*_p_ varied linearly with *ν* according to the linear regression, eqn (8)

8 *I*_p_ = −4.5737*μA* + 208.1227*μν*

where the intercept is −4.5737*μ*, the slope is 208.1227*μ* and the coefficient of correlation is 0.99669.

Similarly, the response of *I*_p_ to the *ν*^1/2^ had also a linear relationship defined by the linear regression, eqn (9)9
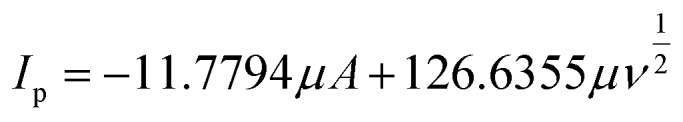
where the intercept is −11.7794*μA*, the slope is 126.6355*μA* V^−1/2^ s^1/2^ and the coefficient of correlation is 0.99621. Notably, the *I*_p_-axis intercept did not pass through the origin, a case that exists for both *I*_p_*vs. v* and *I*_p_*vs. ν*^1/2^ plots, and thus implied some adsorption processes are taking place at the CaCuSi_4_O_10_/GCE modified electrode surface.^45^ It can be concluded that the mass transport during the oxidation process of norfloxacin at the CaCuSi_4_O_10_/GCE modified electrode was controlled by both diffusion and adsorption.

The variation of *E*_p_ to the log *ν* over a scan rate of (5–200 mV s^−1^) was plotted, Fig. 5(e). The plot revealed that *E*_p_varied linearly with log *ν* according to the linear regression, eqn (10)

10 *E*_p_ = 1.0448 + 0.07354 log *ν*

where the intercept is 1.0448, the slope is 0.07354 and the coefficient of correlation is 0.9995. The response of *E*_p_ to log *ν* during the oxidation of norfloxacin was investigated according to the classical equation eqn (3), which relates the peak potential for an irreversible electrode process^46^ to log *ν*. The equation established that *E*_p_ varied linearly with log *ν*. The two simultaneous equations, eqn (3) and (10), can be solved to find out *αn*, which was found to be 0.8. Taking the charge transfer coefficient for irreversible reactions to be from 0.4–0.6,^47^ it implies two electrons were transferred during the oxidation reaction of norfloxacin at the CaCuSi_4_O_10_/GCE modified electrode, and this finding was consistent with previously reported results.^48^

#### 3.3.3 Concentration studies

**Fig. 6:**
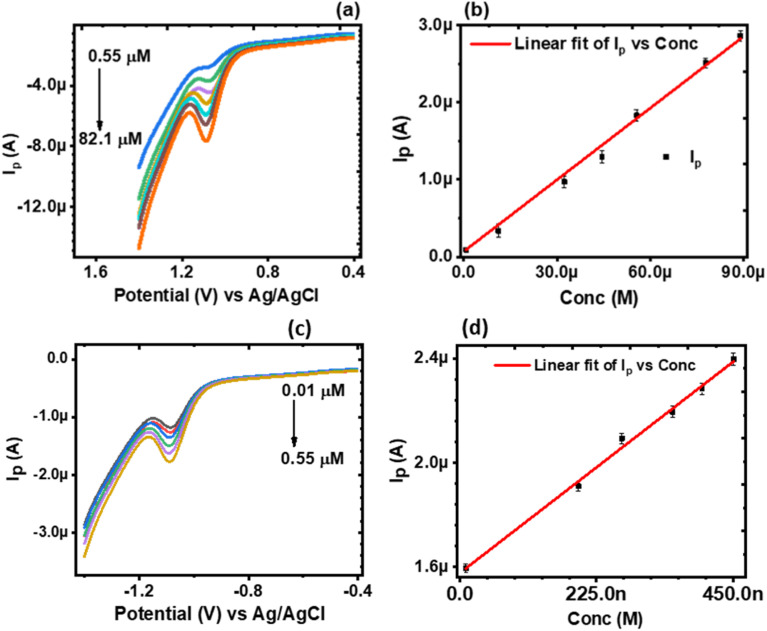
(a) and (c) DPVs of norfloxacin at different concentrations at the CaCuSi_4_O_10_/GCE in 0.1 M PBS (pH 4.5); (b) and (d) the corresponding calibration plot in the linear concentration range 0.55 μM to 82.1 μM and 0.01 μM to 0.55 μM, respectively.

The analytical performance of the CaCuSi_4_O_10_/GCE modified electrode was investigated by electrochemical detection of norfloxacin at various concentration ranges at PBS pH 4.5 using differential pulse voltammetry. A sharp peak corresponding to the oxidation of norfloxacin was observed at 1.089 V, Fig. 6(a) and (c). A plot of *I*_p_ against *C* for norfloxacin concentration was done, Fig. 6(b). It was observed that *I*_p_ varied linearly with *C* according to the linear regression, eqn (11)

11 *I*_p_ = −18.20*μ* + 0.0369*C*

where −18.20*μ* is the intercept, 0.0369 is the slope and the coefficient of correlation is 0.996. The linear relationship was observed in the concentration range 0.55 μM to 82.1 μM. For Fig. 6(d), the relation was:

12 *I*_p_ = −1.60*μ* + 1.8*C*

A linear relationship was also observed in the concentration range 0.01 to 0.55 μM, Fig. 6(d). The limit of detection (LOD) and the limit of quantification (LOQ) of norfloxacin at the CaCuSi_4_O_10_/GCE was calculated from the relationship 3*σ*/*N* and 10*σ*/*N*, correspondingly where *σ* is the standard deviation of the response and *N* is the number of replicates. The LOD was found to be 0.0046 μM and the LOQ was 0.0153 μM. Norfloxacin concentration in contaminated soil can be as high as 9.8 mg kg^−1^, and up to 6.8 μg L^−1^ in urban sewage and surface water,^49^ which are higher concentrations compared to the LOD obtained using the CaCuSi_4_O_10_/GCE electrochemical sensor in this study. The results showed high selectivity of the CaCuSi_4_O_10_/GCE modified electrode towards norfloxacin. A comparison of the present work with the performance of other electroanalytical methods for the detection of norfloxacin reported in the literature is made in Table 1. The current reported method has a comparatively lower detection limit and a wider linear concentration range for the detection of norfloxacin.

**Table 1:** A comparison of the present study with other reported methods for electrochemical determination of norfloxacin

Electrode	Technique	Concentration range (μM)	LOD (μM)	Verified applications	Ref.
MWCNT-CPE/pRGO-ANSAg/Au	DPV	0.03–1.0 and 1.0–50.0	0.016	Pharmaceutical & plasma	43
MWCNTs-TOCT/GCE	CV	0.5–8.0	0.1	Pharmaceutical & urine	50
CuO/MWCNTs/GCE	DPV	1.0–47.7	0.321	None	51
CaCuSi_4_O_10_/GCE	DPV	0.01–0.55 and 0.55–82.1	0.0046	Pharmaceutical	This work

#### 3.3.4 Interference studies

**Fig. 7:**
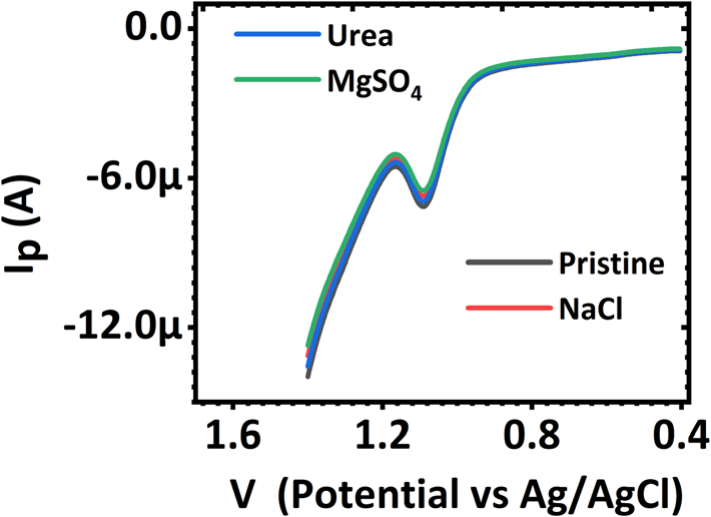
Differential pulse voltammograms of norfloxacin in the presence of different interfering species.

An investigation of the effects of interferents on the electrochemical determination of norfloxacin was carried out using differential pulse voltammetry within the limits of the established linear range, 0.01 to 0.55 μM and 0.55 μM to 82.1 μM. The interference studied was of both organic and inorganic types. In particular, an investigation on the effect of urea, Na^+^, Cl^−^, Mg^2+^, and SO_4_^2−^ on the determination of norfloxacin using the CaCuSi_4_O_10_/GCE modified electrode was performed. The interferents were investigated in concentrations that were 100-fold that of the norfloxacin being detected. The results obtained are shown in Fig. 7. The current response of the norfloxacin oxidation that was obtained as the voltage was swept in the presence of interferents showed that the interference had no significant influence on the electrochemical detection of norfloxacin. The electrochemical system's current response in the presence of the interferents was significantly close to that obtained during the electrochemical detection of norfloxacin alone. This indicated that the interferents did not interfere with the quantitative determination of norfloxacin. The CaCuSi_4_O_10_/GCE electrode retained at least 97.8% of its current response in the presence of the interferents. A relative standard deviation of 2.2%, which is within the limit of the accepted tolerance of 5%, was attained. The CaCuSi_4_O_10_/GCE electrode had good selectivity for the determination of norfloxacin in the presence of interferents.

#### 3.3.5 Reproducibility and stability

Reproducibility of the CaCuSi_4_O_10_/GCE modified electrode was determined by fabricating four identical modified electrodes and applying each of them separately to detect 25.00 μM norfloxacin in 0.1 M PBS at pH = 4.5. The relative standard deviation (RSD) of the anodic peak currents was calculated to be 3.14%, which is less than the accepted tolerance of 5%. One of the electrodes was used to perform the determination on one sample, repeated seven times, and the measurements obtained yielded a relative standard deviation of 4.6%. The same CaCuSi_4_O_10_/GCE modified electrode was used for electrochemical sensing of norfloxacin after 2 weeks and the magnitude of the anodic peak currents obtained for three runs had a standard deviation of 2.37% from anodic peak measurements obtained earlier, which showed the relative stability of the sensor. The determination of norfloxacin using the fabricated CaCuSi_4_O_10_/GCE modified electrode showed high reproducibility and repeatability.

#### 3.3.6 Real sample analysis

The applicability of the CaCuSi_4_O_10_/GCE modified electrode in the electrochemical detection of real samples using DPV was investigated using the pharmaceutical drug formulation Utin-400. The obtained results are shown in Table S1.† Recovery percentages were calculated and found to be in the range of 98.1–102.1% and a bias of less than 2.3% was obtained. The results showed that the CaCuSi_4_O_10_/GCE modified electrode can be used for the efficient determination of norfloxacin in commercial pharmaceutical drug formulations.

## 4 Conclusions

CaCuSi_4_O_10_ was successfully synthesized as confirmed by physical methods of characterization. Electrochemical characterization showed that the CaCuSi_4_O_10_/GCE had a higher current response than the bare GCE. Compared to the bare GCE, the CaCuSi_4_O_10_ had a higher electrochemical surface area and a lower charge transfer resistance. The CaCuSi_4_O_10_ was therefore used for the electrochemical sensing of norfloxacin. The CaCuSi_4_O_10_/GCE modified electrode showed a wider linear concentration range, 0.01 to 0.55 μM and 0.55 μM to 82.1 μM, and LOD, 0.0046 μM, compared to literature reports. It also exhibited satisfactory selectivity and sensitivity towards norfloxacin in the presence of interferents including matrix in pharmaceutical formulation. It can be concluded that the electrochemical quantification of norfloxacin using the CaCuSi_4_O_10_/GCE modified electrode is a viable method since it offers high precision and low detection limits.

## Author contributions

G. Muungani: conceptualization; data curation; formal analysis; investigation; methodology; writing – original draft. W. E. van Zyl: conceptualization; funding acquisition; project administration; resources; supervision; writing – review & editing.

## Conflicts of interest

There are no conflicts of interest to declare.

## Supplementary Material

RA-013-D3RA01702H-s001
