# Supplementary material for: A CaCuSi4O10/GCE electrochemical sensor for detection of norfloxacin in pharmaceutical formulations
Source: RSC Adv. 2023 Apr 25;13(19):12799–808. doi: 10.1039/d3ra01702h (PMC10126821; doi:10.1039/d3ra01702h)
Supplement: RA-013-D3RA01702H-s001 [file RA-013-D3RA01702H-s001.pdf]

Electronic Supplementary Material (ESI) for RSC Advances.
This journal is © The Royal Society of Chemistry 2023

ELECTRONIC SUPPLEMENTARY INFORMATION

$CaCuSi_4O_{10}$/GCE electrochemical sensor for detection of norfloxacin in pharmaceutical formulations

Gregarious Muungani, and Werner E. van Zyl*

School of Chemistry and Physics, University of KwaZulu-Natal, Westville Campus, Durban, 4000, South Africa.

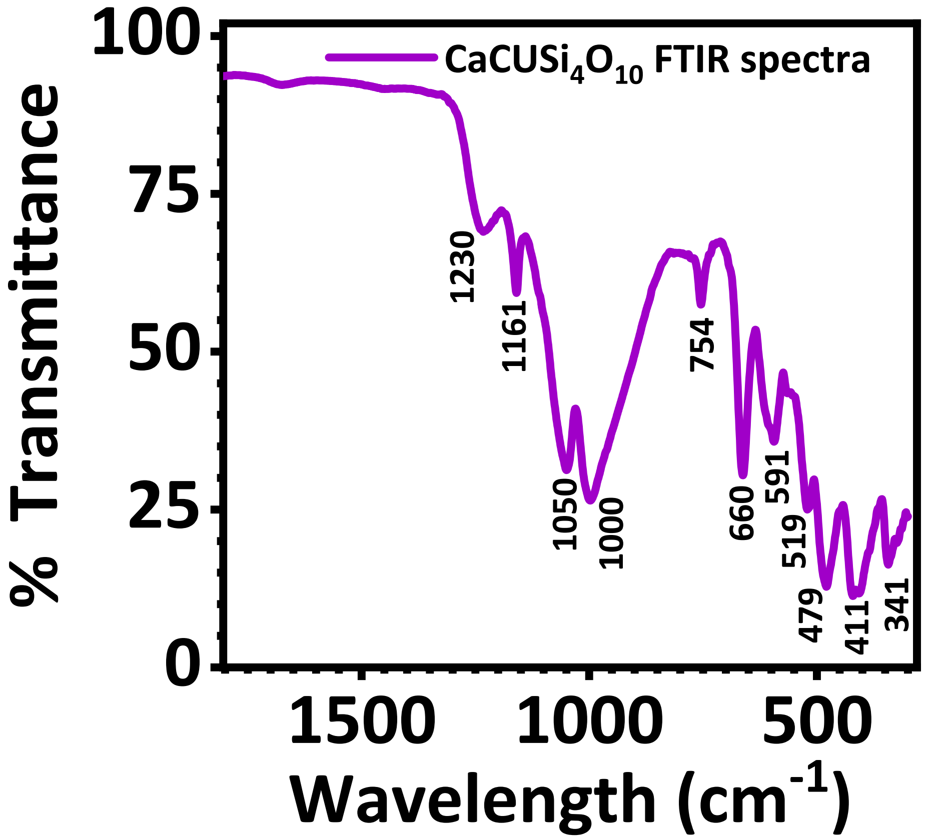

Fig. S1. FTIR spectra of $CaCuSi_4O_{10}$.

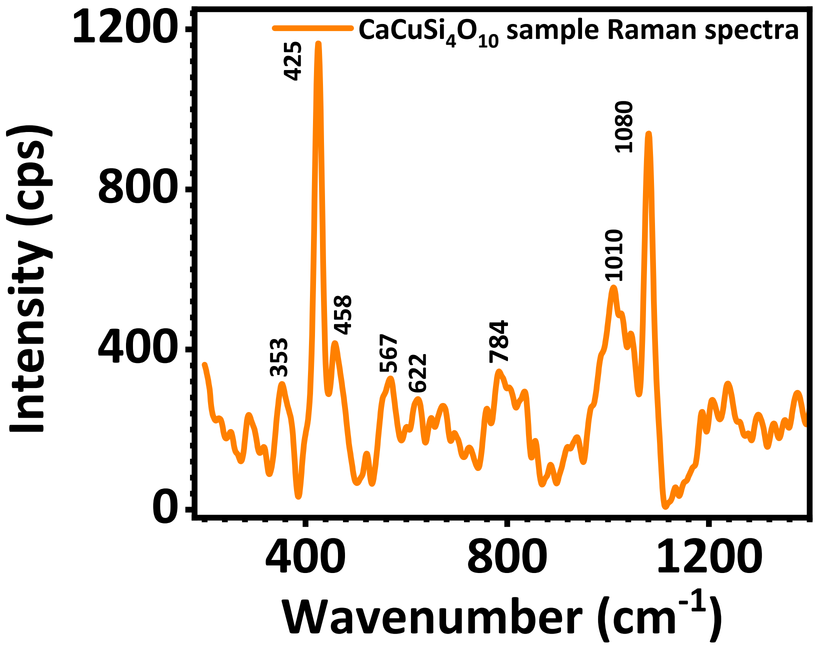

Fig. S2. Raman spectra of $CaCuSi_4O_{10}$.

Table S1

Results obtained for Utin-400 at MnO_2 doped $CaCuSi_4O_{10}$/GCE modified electrode.

S No	Expected (µM)	Detected (µM)	Recovery	Bias (%)	RSD (%) (n=3)
1	10	10.0135	100.1	0.135	0.48
2	20	19.6863	99.9	1.6	0.62
3	30	30.685	102.3	2.28	0.17
4	40	40.586	101.5	1. 5	0.36
5	50	50.692	101.4	1. 4	0.89
6	70	71.62	102.3	0.9	0.53
7	80	78.483	98.1	1.9	1.04
